# Clinical, immunological characterisation and treatment response of patients with syndrome of undifferentiated recurrent fever in Chinese children and adolescents: a single-centre cohort study

**DOI:** 10.3389/fimmu.2026.1683408

**Published:** 2026-02-18

**Authors:** Xiongbin Chen, Wenlu Xing, Yulu Li, Mengyang Yang, Zhou Shu, Huawei Mao

**Affiliations:** 1 Department of Rheumatology and Immunology, National Centre for Children’s Health, Beijing Children’s Hospital of Capital Medical University, Beijing, China; 2 Beijing Key Laboratory of Precision Diagnosis and Treatment of Pediatric Immune Diseases, Beijing, China; 3 Ministry of Education Key Laboratory of Major Diseases in Children, Beijing, China

**Keywords:** autoinflammation, colchicine, FMF, PFAPA, SURF

## Abstract

**Background:**

Syndrome of undifferentiated recurrent fevers (SURF) is a heterogeneous disorder characterised by recurrent fevers and autoinflammation in the absence of a confirmed molecular diagnosis of hereditary recurrent fever (HRF) and periodic fever, adenitis, pharyngitis, aphthous stomatitis (PFAPA) syndrome. The aim of this study is to characterise the clinical and immunological features of SURF patients and to analyse their cytokine signature and treatment patterns.

**Methods:**

Between 2022 and 2024, we enrolled 191 patients who presented to Bei Jing Children’s Hospital, Department of Immunology, with the chief complaint of recurrent fever. Fifty-seven patients met the criteria for SURF, 70 met the criteria for PFAPA and 64 met the criteria for FMF. Baseline data and blood samples were collected from patients at enrolment or at routine clinical visits. Clinical and immunological characteristics and cytokine levels were analysed.

**Results:**

In SURF patients, gastrointestinal symptoms(abdominal pain and vomiting or diarrhoea) were more prominent than in PFAPA patients. However, the difference in gastrointestinal symptoms between SURF patients and FMF patients was not significant. Pharyngitis and cervical adenitis were both seen in SURF and PFAPA patients while the frequency was higher in PFAPA patients. Family history was significantly higher in FMF patients than in SURF patients. The family history was similar between SURF patients and PFAPA patients. Treatment patterns differ between SURF and PFAPA (or FMF) patients. On-demand steroids were more likely prescribed in PFAPA patients, while colchicine was more commonly prescribed in SURF patients. However, no statistically significant differences were found in the prescription of colchicine between SURF and FMF patients. FMF patients were more commonly prescribed on-demand steroids than SURF patients. But SURF patients were more likely prescribed NSAIDs than FMF patients. The B-cell populations and immunoglobulin (Ig) levels (IgG, IgA, IgM and IgE) were similar in both SURF and PFAPA patients (or FMF patients). The proportion of helper T cells (Th cells) (CD3+CD4+) was significantly lower in SURF patients compared to PFAPA patients. However, the proportion of natural killer cells (NK cells) (CD3-CD56+) was significantly higher in SURF patients compared to PFAPA patients. The proportion of cytotoxic T cells (CD3+CD8+) was significantly higher in FMF patients compared to SURF patients. But the proportion and absolute count of natural killer cells (NK cells) (CD3-CD56+) was significantly lower in FMF patients compared to SURF patients. Cytokine levels between SURF and PFAPA patients (or FMF patients) were similar. SURF patients tended to have higher levels of pro-inflammatory cytokines (including IL-1β, IL-6, IL-8, IL-10, TNF-α and IFN-α). Both SURF, PFAPA, and FMF patients showed favourable responses to colchicine treatment.

**Conclusion:**

This study describes the clinical and immunological characteristics of a large cohort of patients with SURF. This suggests us that SURF is a heterogenous disease. However, the clinical and immunological features and treatment options of SURF patients differ from PFAPA and FMF patients.

## Introduction

Fever is one of the most common symptoms in patients that physicians meet in clinical practice. And it mostly caused by infections. However, when fever is recurring or periodic, auto-inflammatory conditions should be considered first (1). Syndrome of undifferentiated recurrent fevers (SURF) was first defined by Broderick et al. in 2020. The features of SURF are recurrent fevers and autoinflammation when a confirmed molecular diagnosis of Hereditary Recurrent Fever syndrome (HRF), and Periodic Fever, Adenitis, Pharyngitis, Aphthous stomatitis syndrome (PFAPA) are excluded (2). It is a group of disorders of innate immunity that cause multisystem inflammation. In addition to recurrent fever, patients with SURF presented with inflammation in the joints, eyes, skin or serosal surfaces. Some patients may experience additional symptoms, including oropharyngeal, gastrointestinal, dermatological and musculoskeletal manifestations (3).

Over the past 5 years, more and more children have been diagnosed with SURF as doctors’ awareness of these rare conditions has increased and sequencing technology has advanced and become available. However, 60–80% of them lack a molecular or genetic diagnosis (4, 5). The most common diagnosis for paediatric patients with recurrent fevers and autoinflammation is PFAPA (6).

Before the concept of SURF was proposed, many patients may be misdiagnosed with PFAPA although they did not fulfil the diagnostic criteria of PFAPA, they also have been called as atypical PFAPA (2). According to the Yalcinkaya-Ozen criteria, patients presenting with recurrent fever and abdominal symptoms may be misdiagnosed with familial Mediterranean fever (FMF) (7). These patients exhibit symptoms that are similar to those of PFAPA and FMF, but with limited associated symptoms of aphthous stomatitis, pharyngitis, and/or adenopathy. It is becoming recognised that these patients may represent a distinct clinical entity, which has come to be known as SURF. A study by Şengül Çağlayan et al. showed that the current Eurofever/PRINTO criteria are unable to distinguish between patients with SURF and FMF (8). The study by Macaraeg et al. revealed differences in treatment regimen selection and cytokine profiles between SURF and PFAPA patients (9). Another study has also shown that the immune signatures of patients with PFAPA and SURF are different. These suggest that SURF and PFAPA(or FMF) are clinically distinct entities.

The clinical features, immunological characteristics and treatment of SURF in children are currently poorly understood. It is important for general paediatricians, paediatric rheumatologists and even adult clinical immunologists to recognise SURF, especially to better differentiate SURF from PFAPA and FMF. The aim of this study is to better characterize SURF patients in a cohort study.

## Materials and methods

### Patients and subjects

Patients who were diagnosed with recurrent fever syndromes between January 2022 and December 2024 at Bei Jing Children’s Hospital, Immunology Department were enrolled. It is a prospective study. Between January 2022 and December 2024, we continuously enrolled patients who might be diagnosed with recurrent fever syndromes. Demographic data, clinical characteristics, laboratory outcomes and response to treatment data were documented by medical file screening. Patients met the following criteria to be defined as SURF: 1) Patients with at least three episodes in the past of stereotypical recurrent fevers (without evidence of infections, malignancy and immunodeficiency). 2) Asymptomatic between episodes. 3) Absence of genetically defined autoinflammatory disorders associated with HRFs (such as MEFV, MVK, TNFRS1A, NLRP3) (4). 4) Patients’ episodes could not be explained by PFAPA. 5) Absence ofyclic neutropenia. Diagnosis of PFAPA syndrome established according to modified Marshall criteria (10), regularly recurrent fever, absence of upper respiratory tract infection and at least one of the following clinical signs: pharyngitis, aphthous stomatitis or cervical adenitis, completely asymptomatic intervals between febrile episodes, normal growth and development. The diagnosis of FMF was made based on clinical findings and genetic testing. Patients should meet the “Yalcinkaya-Ozen” criteria (7) (it requires recurrent (≥3) attacks with at least 2 of the following 5 features: fever lasting between 12 and 72 hours, abdominal pain, chest pain, arthritis, and a positive family history for FMF)and mutations in the MEFV gene were also detected.

Patients were then treated by paediatric rheumatologists as soon as they were enrolled. Response to treatment was defined as: i) complete response (absence of clinical manifestations and elevation of acute phase reactants between the episodes), ii) partial response (persistence of some clinical manifestations with reduction of at least 50% in fever episodes), iii) Treatment failure was defined as no change in symptoms (or even worsening) with treatment.

### Genetic analysis

Whole exon sequencing (WES) and Sanger sequencing were performed in patients with suspected recurrent fever syndromes after obtaining the consent of parents or guardians. DNA extraction was performed using the Axygen DNA Mini Kit (Axygen, China) and genomic DNA was extracted from the patient’s whole blood for sequencing. Once suspected mutations were obtained, the pathogenicity of each variant was calculated according to American College of Medical Genetics and Genomics(ACMG) guideline criteria. Genetic variants were classified as being pathogenic, likely pathogenic, of uncertain significance, likely benign or benign (11, 12). Patients with genes associated with HRFs were excluded from the SUFR cohort if they were considered to be pathogenic or likely pathogenic variants.

### Quantification of cytokines

Serum samples from patients were isolated from peripheral blood collected in vacutainers containing sodium heparin and stored at -80 °C for cytokine detection. Serum cytokine analyses were performed on a bead-based immunoassay (LEGENDplex™ Human Inflammation Panel, BioLegend) according to the manufacturer’s protocol.

### Evaluation of immunological function

Lymphocyte subsets and immunoglobulins G, A, M and E were analysed to assess immunological function. Immunoglobulins G, A and M were detected by nephelometry. Immunoglobulin E was detected by UniCAP (Pharmacia, Uppsala, Sweden). Peripheral blood samples were collected from patients at enrolment or at routine clinical visit (The patient may not have any symptoms during the blood collection). Serum and plasma samples were stored at -80 °C within 120 minutes of collection. Lymphocyte subsets were detected by a flow cytometry (BD Biosciences). Briefly, peripheral blood was stained with appropriate fluorochrome-labelled monoclonal antibodies or isotype-matched control antibodies for 30 minutes in the dark. The red blood cells were then lysed and the cells washed twice with FACS buffer (PBS buffer contained 3%FBS(Foetal bovine serum)). The validated antibodies included CD3, CD4, CD8, CD19, CD27, CD56 and IgD which were purchased from Biolegend.

### Statistical analysis

Statistical analysis was performed with the IBM Statistical Package for the Social Sciences (SPSS) V.26. GraphPad Prism 8.0 is used to draw figures. Categorical variables were described as frequencies and percentages. Numeric variables were reported as the median and 1st and 3rd quartiles (IQR) or mean ± standard error of mean (SEM). To compare dichotomous variables with interval or ordinal variables, the Mann-Whitney U test was performed. A two-tailed p-value of 0.05 was considered to be statistically significant.

## Result

### Demographic and clinical features of patients with SURF, FMF and PFAPA

Between 2022 and 2024, a total of 191 patients were included in our cohort. Of these patients, 57 met the criteria for SURF, 70 met the criteria for PFAPA and 64 met the criteria for FMF. Clinical and demographic features of patients are shown in **Table 1**. The gender ratios of children with SURF, FMF and PFAPA are similar. The median age at symptom onset and diagnosis were similar for SURF, FMF and PFAPA patients. The maximum temperature during an episode was also similar for these patients (P > 0.05). All patients were asymptomatic during the afebrile period and showed normal growth and development. Family history for recurrent fever was reported in only 3 patients (5.3%) of the SURF patients and in 8 patients (11.4%) of the PFAPA patients. While the family history was reported in 20 patients (31.3%) in FMF patients, which was significantly higher than SURF patients(P = 0.0001).

**Table 1:** Demographic and clinical features of patients with SURF,PFAPA and FMF at baseline.

Variables	FMF (n=64)	P-value (FMF VS SURF)	SURF (n=57)	PFAPA (n=70)	P-value (PFAPA VS SURF)
Female, N (%)	22 (34.4%)	0.78	21 (36.84%)	37 (52.86%)	0.077
Age at onset, median years (IQR)	5.0 (1.37-7.29)	0.081	2.17 (1.00-5.6)	3.34 (1.21-5.75)	0.42
Age at diagnosis, median years (IQR)	7.25 (2.1-8.5)	0.249	5.00 (2.30-7.75)	4.96 (2.65-7.04)	0.95
Diagnostic delay, median years (IQR)	1.125 (0.33-2)	0.606	1 (0.59-2.00)	1 (0.62-2.00)	0.92
T max (°C, IQR)^1^	39.8 (39-40)	0.8	40 (39.8-40)	40 (39.5-40)	0.66
Duration of episodes (days, IQR)	3.75 (2.5-5.5)	0.872	4 (3.125-4.5)	4 (3.38-4.5)	0.67
Frequency of the febrile episode (weeks, IQR)	3.75 (2.5-4)	0.946	3.5 (3-4)	4 (3-4)	0.24
Family history, N (%)	20 (31.3%)	**0.0001**	3 (5.3%)	8 (11.4%)	0.34
Pharyngitis, N (%)	12 (18.8%)	**0.0001**	34 (59.6%)	66 (94.3%)	**<0.0001**
Cervical adenitis, N (%)	8 (12.5%)	0.065	15 (26.3%)	40 (57.1%)	**0.001**
Oral aphthosis, N (%)	10 (15.6%)	0.18	15 (30%)	25 (35.7%)	0.339
Abdominal pain, N (%)	22 (34.4%)	0.9	19 (33.3%)	2 (2.9%)	**<0.0001**
Rash, N (%)	8 (12.5%)	0.214	3 (5.3%)	1 (1.4%)	0.325
Arthralgia, N (%)	8 (12.5%)	0.1	2 (3.5%)	2 (2.9%)	1.00
Vomiting or diarrhoea, N (%)	10 (15.6%)	0.283	5 (8.8%)	1 (1.4%)	0.089
Treatment, N (%)					
Colchicine	32 (50%)	1	28 (49.1%)	21 (30%)	**0.03**
On demand steroids	14 (21.9%)	**0.003**	2 (3.5%)	13 (18.57%)	**0.011**
On demand NSAIDs^2^	16 (25%)	**0.035**	25 (43.9%)	30 (42.86%)	1.00
Thalidomide	2 (3.1%)	1	2 (3.5%)	2 (2.86%)	1.00
Tonsillectomy	0	–	0	4 (5.71%)	0.127

"Bold values" indicate that the differences are statistically significant.

Patients with PFAPA had significantly higher rates of pharyngitis and cervical adenitis than patients with SURF. Interestingly, the frequencies of pharyngitis was significantly higher in SURF patients than FMF patients. While the frequencies of cervical adenitis and oral aphthosis in FMF patients were not significant difference compared with SURF. Gastrointestinal symptoms (abdominal pain, vomiting or diarrhoea) were more prominent in SURF patients than in PFAPA patients. But the difference in gastrointestinal symptoms(abdominal pain and vomiting or diarrhoea) between SURF patients and FMF patients was not significant. Rash and arthralgia were both seen in SURF, PFAPA and FMF patients, with no obvious difference between them.

To investigate the differences in treatment patterns between SURF, PFAPA and FMF patients in our cohort. Non-steroidal anti-inflammatory drugs (NSAIDs) and colchicine were most commonly required in these three groups of patients. On-demand steroids were more commonly prescribed in PFAPA patients than SURF patients, while colchicine was more commonly prescribed in SURF patients. However, no statistically significant differences were found in the prescription of colchicine between SURF and FMF patients. Similar to PFAPA patients, FMF patients were more commonly prescribed on-demand steroids than SURF patients. But SURF patients were more likely required NSAIDs than FMF patients.

### 1.T max: Highest body temperature at onset of diseases. 2. NSAIDs: Non-steroidal anti-inflammatory drugs.Immunological features of the SURF and PFAPA patients

To analyse the immunological characteristics of SURF, PFAPA and FMF patients, we analysed their lymphocyte subpopulation and immunoglobulins G, A, M and E. The immunological characteristics of the patients are listed in **Table 2**. We observed that the absolute count of lymphocytes, the B lymphocyte subpopulations(Memory B cells(IgD-CD27+) and Naïve B cells(IgD+CD27-)) and the immunoglobulin (Ig)G, IgA, IgM and IgE levels were similar in SURF and PFAPA patients. The same results were also observed in SURF and FMF patients. The proportion of helper T cells (Th cells) (CD3+CD4+) was significantly higher in PFAPA patients compared to SURF patients. However, the proportion of natural killer cells (NK cells) (CD3-CD56+) was significantly lower in PFAPA patients compared to SURF patients. Interestingly, the proportion of cytotoxic T cells (CD3+CD8+) was significantly higher in FMF patients compared to SURF patients. But the proportion and absolute count of natural killer cells (NK cells) (CD3-CD56+) was significantly lower in FMF patients compared to SURF patients. These differences suggest that SURF is likely to be a pathologically independent entity from PFAPA and FMF and is not simply an atypical form of PFAPA or FMF.

**Table 2:** Immunological characteristics of SURF, FMF and PFAPA patient.

Lymphocyte subpopulation, N/total% (cells/μL)	FMF	P-value (FMF *vs* SURF)	SURF	PFAPA	P-value (PFAPA *vs* SURF)
T cells, median(IQR)	2331(1228-2800)	0.238	2568(1755.25-3468.25)	2427(2106-3155)	0.53
T cells, N/total%	72.4%	0.05	66.7%	69.5%	0.079
Th cells (CD3+CD4+), median(IQR)	1171(609-1317)	0.19	1222.5(899.5-1968.25)	1497(1040-1845)	0.31
Th cells (CD3+CD4+), N/total%	35%	0.935	33.1%	36.4%	**0.012**
Cytotoxic T cells (CD3+CD8+), median(IQR)	826(540-1374)	0.512	816.5(561.25-1032.5)	832(667-957)	0.45
Cytotoxic T cells (CD3+CD8+), N/total%	27.3%	**0.0001**	20.3%	22.5%	0.15
B cell counts (CD19+), median(IQR)	415(216-733)	0.094	594(379.75-1044)	632(498-1111)	0.34
B cell counts (CD19+), N/total%	15.2%	0.105	17.6%	18%	0.98
Memory B cells(IgD-CD27+), median(IQR)	89(35-161)	0.817	125(7-149)	112(68.75-147)	0.9
Memory B cells(IgD-CD27+), N/total%	18.9%	0.052	12.6%	19.05%	0.4
Naïve B cells(IgD+CD27-), median(IQR)	97.5(76-454)	0.354	208(66-476)	323(184.5-738)	0.8
Naïve B cells(IgD+CD27-), N/total%	34.2%	0.105	70.9%	53.15%	0.8
NK cell counts (CD3-CD56+), median(IQR)	279.5(143-423)	**0.006**	448(286.75-779.25)	324(231-513)	0.2
NK cell counts (CD3-CD56+), N/total%	9.5%	**0.001**	12.1%	10.55%	**0.047**
Immunoglobulin					
IgG (g/L), median (IQR)	10(6.33-13.53)	0.261	9.3(7.42-10.85)	9.3(7.5-10.53)	0.73
IgA (g/L), median (IQR)	2.1(1.23-3.35)	0.117	1.49(0.79-2.2)	1.46(0.81-1.97)	0.54
IgM (g/L), median (IQR)	1.02(0.81-1.56)	0.783	1.02(0.82-1.39)	1(0.76-1.28)	0.41
IgE (IU/ML), median (IQR)	58.2(21.05-286.2)	0.177	36.6(19.5-80.05)	29.8(10.1-109)	0.54

"Bold values" indicate that the differences are statistically significant.

### Th cell, helper T cell; NK cell, natural killer cell; Ig, immunoglobulins.Cytokine levels in patients with SURF and PFAPA

**Figure 1:**
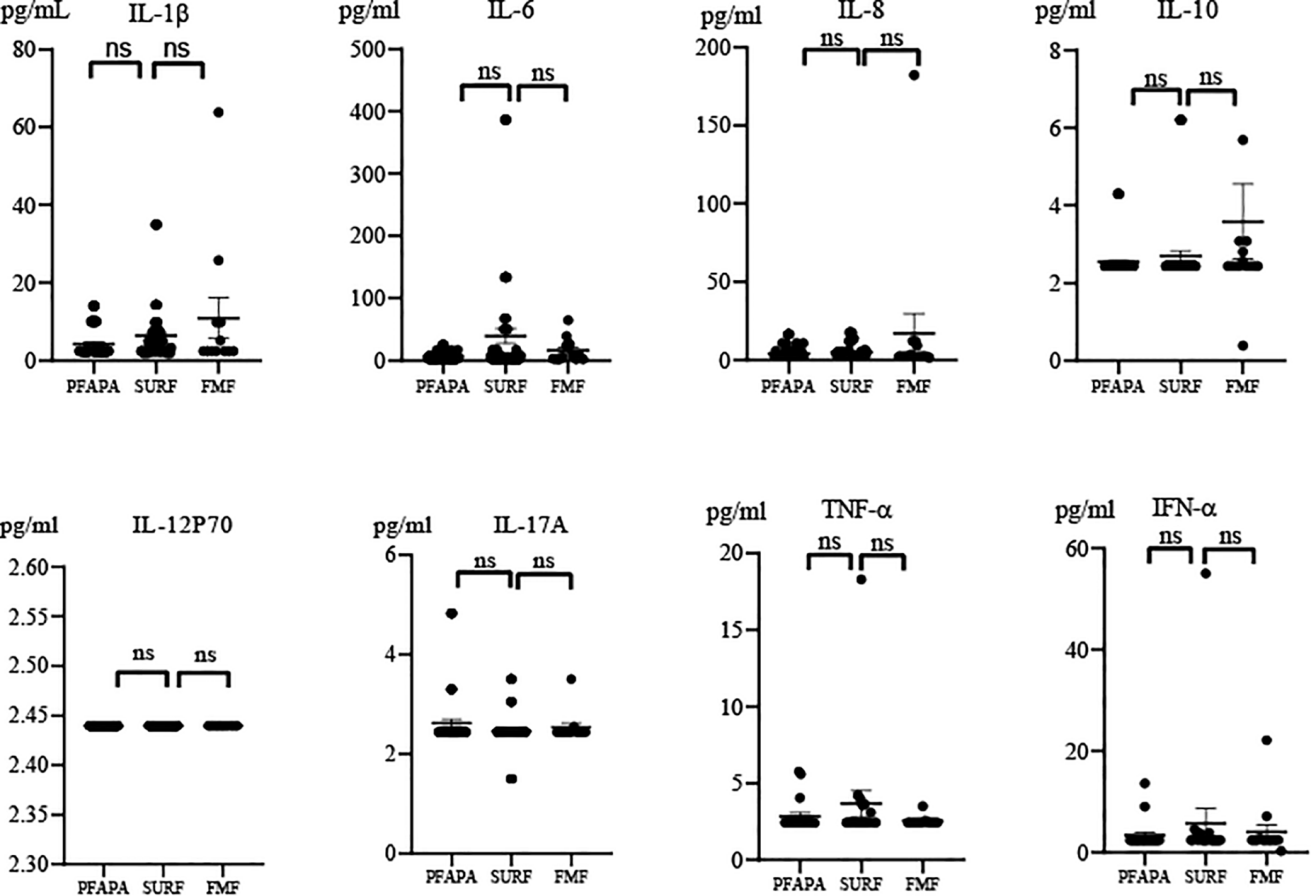
Serum cytokine levels in SURF, PFAPA and FMF patients. Data from SURF, PFAPA and FMF patients were pooled, respectively (ns, no statistical difference; Mann-Whitney U test). Values were represented as mean ± SEM.

In order to investigate the differences in inflammatory factors between SURF and PFAPA patients, we performed cytokine detection in patients. Peripheral blood samples were collected from patients at the time of enrolment or at a routine clinical visit, which was not related to the onset of symptoms. As shown in **Figure 1**, the difference in cytokine levels between SURF and PFAPA patients (or FMF patients) was not statistically significant. Interestingly, there was a tendency for SURF patients to have higher levels of pro-inflammatory cytokines, particularly interleukin (IL)-1β, IL-6, IL-8, IL-10, tumour necrosis factor-α(TNF-α) and interferon-α (IFN-α) than PFAPA patients. However, the IL-1β, IL-8 and IL-10 levels seemed to be higher in FMF patients when compared to SURF patients. While IL-6, TNF-α and IFN-α with a tendency to be higher in SURF patients than FMF patients. The IL-17A and IL-12P70 levels were not higher in SURF patients.

### Efficacy of colchicine in SURF patients

**Figure 2:**
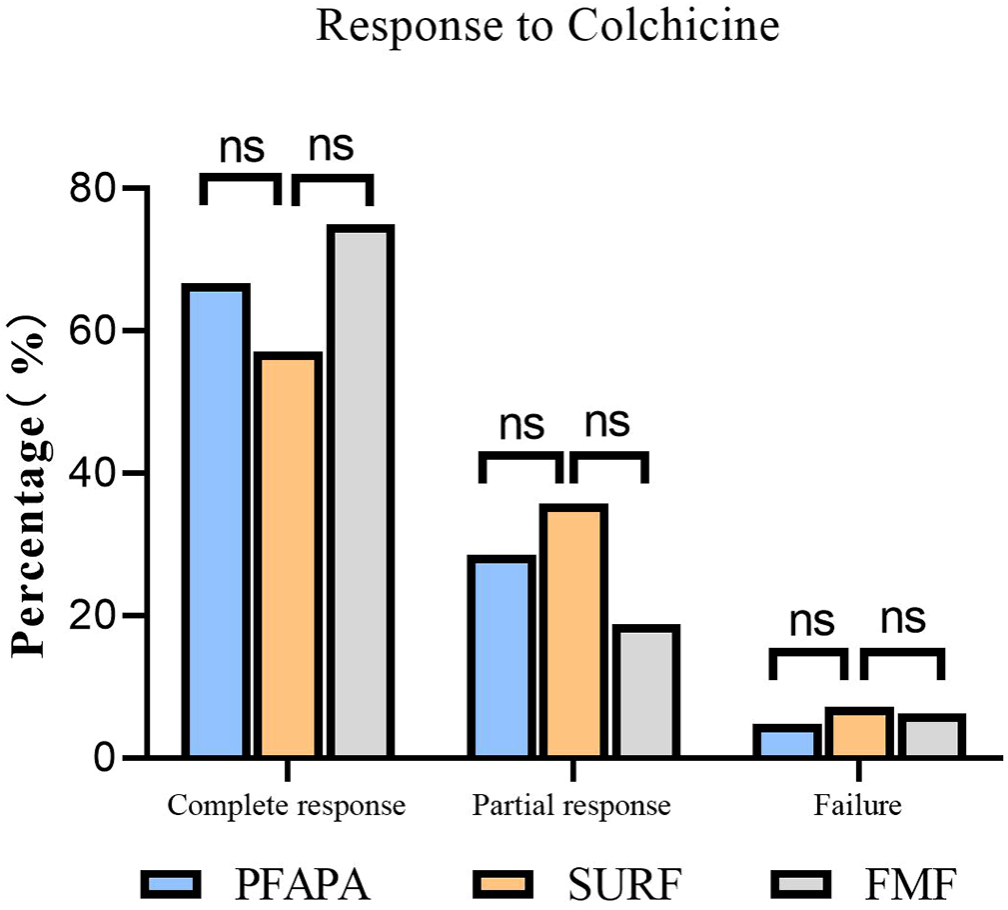
Colchicine efficacy in SURF, PFAPA and FMF patients. Complete response: absence of clinical manifestations and elevation of acute phase reactants between the episodes. Partial response: persistence of some clinical manifestations with reduction of at least 50% in fever episodes. Treatment failure: no change in symptoms (or even worsening) with treatment. (ns, no statistical difference).

The treatments and response rates for patients are presented in **Figure 2**. In SURF patients, twenty-eight patients received colchicine. Of these patients,16 (57.14%) had a complete response. Ten patients (35.71%) had a partial response, which means these patients had a reduction of at least 50% in fever episodes. Two patients (7.15%) did not respond to colchicine and it was considered that they should stop taking colchicine and switch to thalidomide. In patients who taking colchicine, one patient had abdominal pain, nausea and dizziness, and one patient had abdominal pain and constipation. However, these symptoms were not serious and they continued to take colchicine. In PFAPA patients, 21 patients received colchicine. Of these patients,14 (66.67%) had a complete response. Six patients (28.57%) had a partial response and one patient (4.76%) did not respond to colchicine and switch to thalidomide. And in FMF patients, 32 patients received colchicine. Twenty-four patients (75%) had a complete response. Six patients (18.75%) had a partial response and two patients(6.25%) did not respond to colchicine.

## Discussion

Fever is one of the most common signs in clinical practice. There are many conditions that can have febrile manifestations, such as infections, tumours and autoimmune diseases, and auto-inflammatory disorders (13). Syndrome of undifferentiated recurrent fever (SURF) is most commonly seen in auto-inflammatory diseases (AIDs). It is difficult to diagnose SURF in a large population, there is a lack of criteria for the diagnosis of SURF, and there can be considerable overlap in the clinical presentation of different diseases. The diagnosis of SURF mainly depends on clinical symptoms, so there is an inevitable delay in diagnosis. Many patients have seen five or more hospital specialities before a diagnosis is made (14). It is a significant clinical problem and a definitive diagnosis is difficult to make in many patients, which means that the best approaches to treatment are not known. In the present study, we describe a cohort of SURF patients that may distinct from PFAPA and FMF patients.

Distinguishing between SURF and PFAPA is challenging, especially in the absence of widely accepted diagnostic criteria for PFAPA. There are many classification criteria for the diagnosis of PFAPA. The diagnostic criteria for PFAPA was initially proposed by Marshall et al. (15), and then was modified by Thomas et al. in 1999 (10). In 2018 Vanoni et al. describe a new set of criteria, setting 6 years of age as the upper limit for onset of disease (16). In 2019 Takeuchi et al. set 5 years of age as the upper limit for onset of disease (17). Most recently, Gattorno et al. defined updated diagnostic criteria for PFAPA that do not mention an age limit for disease onset (18). However, none of these criteria have been universally adopted. In our study, the most common symptoms in SURF patients were pharyngitis (59.6%), oral aphthosis (30%), abdominal pain (33.3%) and cervical adenitis (26.3%), while the most common symptoms in PFAPA patients were also pharyngitis (94.3%), oral aphthosis (35.7%) and cervical adenitis (57.1%).However, pharyngitis and cervical adenitis were more common in PFAPA patients than in SURF patients. While gastrointestinal symptoms(abdominal pain and vomiting or diarrhoea) were more common in SURF patients. This suggests to us that there may be differences in clinical manifestations between SURF and PFAPA patients, that is, gastrointestinal symptoms are more prominent in SURF patients. These findings are consistent with previous studies by Irene et al (19).

The diagnostic criteria for FMF are the Yalcinkaya-Ozen criteria (7). However, it is difficult to distinguish SURF patients with obvious abdominal symptoms from FMF patients. The research conducted by Marci Macaraeg et al. indicates that 40% of SURF patients experience fatigue or gastrointestinal symptoms (9). A study that attempted to distinguish diseases such as FMF from SURF by the Eurofever/PRINTO criteria found that the sensitivity of the Eurofever/PRINTO classification criteria was low. The authors argue that, in real life, patient-based evaluation is more accurate than standard classification criteria (8). In our study, the gastrointestinal symptoms were not significant in either the SURF or FMF patients. Family history may be the difference between SURF and FMF, so genetic testing for SURF patients may help with the diagnosis.

In our study, non-steroidal anti-inflammatory drugs (NSAIDs) and colchicine were most commonly used in both SURF patients and PFAPA patients. None of the patients in the SURF group were undergoing tonsillectomy. This may because surgical therapies are largely driven by parental preference. The study by Nienke et al. suggests that most patients were treated with steroids, NSAIDs and colchicine (20). Our results support this, although there were some differences in steroids and tonsillectomy. The treatment patterns between SURF patients and PFAPA patients were different. On-demand steroids were more commonly prescribed in PFAPA patients (P = 0.011), while colchicine was more commonly prescribed in SURF patients (P = 0.03). These were similar to previous studies (9). Colchicine is recommended for the treatment of FMF. In many studies, the effect of colchicine therapy has been well established in randomized trials (21, 22). Approximately half of the patients in our study received colchicine treatment. This may be due to the convenience of taking the medication. Some parents believed it was better to take NSAIDs when symptoms occurred than to take the medication daily. We will provide different treatment suggestions and then the guardians will choose the one they think is the most effective for the child.

Interestingly, there were no statically significant difference between SURF patients and FMF patients in the prescription of colchicine. FMF patients were more commonly prescribed on-demand steroids than SURF patients. But SURF patients were more likely required NSAIDs than FMF patients. None of the patients in our study received anti-IL1 therapy. This may be because the anti-IL-1 antagonist will not be approved for use in children in China until 2023. Anti-IL-1 therapy is recommended for both SURF, PFAPA and FMF patients, and is more likely to be prescribed in SURF patients (9).In our study, twenty-eight SURF patients received colchicine. Of these patients, sixteen patients (57.14%%) had a complete response to colchicine and ten patients (35.71%) had a partial response, while two patients(7.15%) did not respond to colchicine. Treatment responses to colchicine in our study were similar to the study by Sutera et al(complete response rate, partial response rate and failure rate were 64.6%,18.8% and 16.6%,respectively) (23). In PFAPA patients, the complete response rate, partial response rate and failure rate were 66.67%,28.57% and 4.76%,respectively.The study by Veronica Gomez-Caverzaschi et al. also suggested that the complete responses were achieved in 40.2%, and 42.3% had a partial response in PFAPA and undifferentiated autoinflammatory diseases patients (24). In other studies, the complete response rate to colchicine in PFAPA patients was 60%, and the partial response rate was 19% (25, 26). Oral colchicine has been found to be very effective in reducing the frequency of attacks and preventing renal amyloidosis in FMF. Most patients require a dose of 1–2 mg of colchicine to control their attacks. Long-term oral colchicine therapy was established as quite safe, with only mild and infrequent side effects (27–29). In our study, thirty-two FMF patients received colchicine. Twenty-four patients (75%) had a complete response, six patients (18.75%) had a partial response and two patients did not respond to colchicine. This is consistent with the findings of Ori Goldberg et al (30). These further suggest that SURF patients may benefit from colchicine therapy. Thalidomide exerts its therapeutic effect by inhibiting inflammatory factors such as tumour necrosis factor-α (TNF-α) and IL-6, interfering with the vascular endothelial growth factor (VEGF) signalling pathway, and influencing the functions of immune cells. For patients who do not respond to colchicine, thalidomide can be chosen as a treatment option.

T cells play an important role in cell-mediated immunity. Different T cell subsets can be identified by their cell surface biomarkers. Th cells (CD3+CD4+) support the immune process by assisting other lymphocytes, including the maturation of B cells and the activation of cytotoxic T cells and macrophages. Cytotoxic T cells (CD3+CD8+) recognise their target cells by binding to major histocompatibility complex class I antigens and destroying infected and tumour cells (31).Flow cytometric analysis showed that the leukocyte populations in SURF patients were different from PFAPA and FMF patients. In our study, we found that immunological characteristics of SURF and PFAPA patients were different. The proportion of Th cells(CD3+CD4+) was significantly lower in SURF patients compared to PFAPA patients. While SURF patients had a trend towards increased NK cells when compared to PFAPA patients. These were similar to previous studies showing that the leukocyte populations of SURF patients differed from those of PFAPA patients, with decreased CD3+ T cells (especially the CD4+ subset) and increased NK cells in Tonsillar cells (19). In PFAPA patients, flow cytometry evaluation showed an increase in CD8+ T cells, CD19+ B cells and CD19+CD20+CD27+CD38-memory B cells compared to patients with infectious pharyngitis (32). In our study, peripheral blood B lymphocytes (including memory B cells (IgD-CD27+) and Naïve B cells(IgD+CD27-)) and IgG, IgA, IgM and IgE levels were similar in both SURF and PFAPA patients in our study. However, Luu et al. examined tonsils from SURF and PFAPA patients after tonsillectomy and found that tonsils from PFAPA patients had a significantly larger memory B cell population (19). This suggests to us that measuring localised lymphocytes is more useful than measuring peripheral lymphocytes. And it also suggests to us that SURF is also an entity that is independent of the infectious disease. There is no significant difference in humoral immunity between SURF and PFAPA patients, and we cannot distinguish them on the basis of differences in immunoglobulins. Although the absolute count of T cells, cytotoxic T cells and Th cells were similar in both SURF patients and PFAPA patients. But the proportion of cytotoxic T cells (CD3+CD8+) was significantly higher in FMF patients compared to SURF patients. But the proportion and absolute count of natural killer cells (NK cells) (CD3-CD56+) was significantly lower in FMF patients compared to SURF patients. This suggests that, like PFAPA and FMF, SURF is a type of autoinflammatory disease. The differences observed in T lymphocyte subsets suggest that SURF has a distinct pathogenic mechanism to FMF or PFAPA. It is possible that SURF is caused by an as-yet-unidentified pathogenic gene. The difference in the proportion of T cell subsets is sufficient enough to suggest that SURF and PFAPA(or FMF) patients have different immunological characteristics and more likely different pathogenesis.

In this study, we also evaluated peripheral cytokines in SURF, PFAPA and FMF patients. We found that peripheral cytokines between SURF and PFAPA (or FMF) patients were similar. However, we noticed that SURF patients appeared to have higher levels of pro-inflammatory cytokines compared to PFAPA patients, including IL-1β, IL-6, IL-8, IL-10, TNF-α and IFN-α. This may be another immunological feature of SURF and PFAPA patients. However, the IL-1β, IL-8 and IL-10 levels seemed to be higher in FMF patients when compared to SURF patients. While IL-6, TNF-α and IFN-α with a tendency to be higher in SURF patients than FMF patients. To date, only a small number of studies have investigated the differences in cytokines between SURF and PFAPA(or FMF) patients. Luu et al. performed cytokine analysis of tonsils from SURF and PFAPA patients who underwent tonsillectomy and found that IL-1β levels were higher in SURF patients than in PFAPA patients (19). Macaraeg et al. also evaluated cytokine levels in SURF and PFAPA patients and found that the cytokine levels between PFAPA and SURF patients were similar, while macrophage inflammatory protein 1β(Mip-1β) was significantly higher in PFAPA patients (9). IL-1β is an important pro-inflammatory factor. When various signals trigger the inflammasome, it binds to caspase-1, leading to activation of caspase-1, which cleaves pro-IL-1β and promotes IL-1β production (33). The study by Serena Palmeri et al. showed that analysing the function of the pyrin inflammasome can distinguish between SURF, FMF and PFAPA patients. This suggests that the pyrin inflammasome is involved in the pathophysiology of SURF (34). Anti-IL-1 therapy may be an effective treatment for SURF patients, as their IL-1β levels are higher than PFAPA patients. Macaraeg et al. demonstrated that 71% of the patients who received IL-1 inhibition had an improvement in their symptoms (9). These findings are further evidence that the diagnosis and treatment of SURF patients is different from PFAPA and FMF patients.

Our study has some limitations that need to be acknowledged. Firstly, this is a single-centre study with a small sample size. We need to further validate our results in a larger population. Additionally, serum samples were collected only during intermissions of disease episodes. So we don’t know the cytokine levels at the onset of the disease. Unfortunately, for political reasons, none of our patients received anti-IL-1 therapy, so we were unable to assess the safety and efficacy of biological agents. In other studies, a proportion of patients received anti-IL-1 therapy and had a good response.

In conclusion, SURF is a heterogenous disease of the autoinflammatory disease spectrum. The clinical and immunological features and treatment options of SURF patients differ from PFAPA and FMF patients. Overall, this suggests to us that SURF and PFAPA (or FMF) patients are clinically distinct entities with distinct pathophysiologic mechanisms. Differences in T lymphocytes may be an important indicator for distinguishing SURF from FMF or PFAPA.A large sample, multi-centre, prospective study is needed to further understand the specific characteristics of SURF.

## Data Availability

The original contributions presented in the study are included in the article/supplementary material. Further inquiries can be directed to the corresponding author.
